# Effectiveness and safety of anakinra in gouty arthritis: A case series and review of the literature

**DOI:** 10.3389/fmed.2022.1089993

**Published:** 2023-01-12

**Authors:** Sicylle Jeria-Navarro, Alejandro Gomez-Gomez, Hye Sang Park, Enrique Calvo-Aranda, Hector Corominas, Maria Antonia Pou, Cesar Diaz-Torne

**Affiliations:** ^1^Rheumatology Department, Hospital Universitari de la Santa Creu i Sant Pau, Barcelona, Spain; ^2^Rheumatology Department, Hospital Universitari Vall d'Hebron, Barcelona, Spain; ^3^Crystal-induced Arthritis Study Group, Spanish Society of Rheumatology (GEACSER), Madrid, Spain; ^4^Rheumatology Department, Hospital Universitario Infanta Leonor, Madrid, Spain; ^5^EAP Encants, SAP Dreta de Barcelona, ICS, Barcelona, Spain

**Keywords:** gout, anakinra, IL-1 inhibition, gout flare, biological therapies, gouty arthritis, IL-1 blocking agents, IL-1 blockade

## Abstract

**Background:**

Gout is the most common type of inflammatory arthritis. Nonsteroidal anti-inflammatory drugs, corticosteroids, and colchicine are the first-line agents, although they are contraindicated in many patients. Blockade of IL-1 with anakinra can be an alternative.

**Objective:**

To present a case series of 10 difficult-to-treat gout patients treated with anakinra and perform a scoping review of the effectiveness and safety of anakinra in gout patients.

**Methods:**

A total of 1,519 citations were screened. The reviewers ran a two-stage screening process by title/abstract and full-text reading. Thirty-eight articles finally met the selection criteria and were included for data extraction and synthesis. Experience in difficult-to treat and complex clinical scenarios, such as active infection, hemodialysis, and transplantation, were specifically described.

**Results:**

The study sample comprised 551 patients, from whom 648 flares were finally analyzed. The mean age was 57.9 years, and 82.9% were men. The clinical presentation was polyarticular in 47.5% and tophaceous in 66.9%. Sixty-five patients with an active infection, 41 transplanted patients and 14 in haemodyalisis treated with anakinra are described. More than half of the patients had >1 associated comorbidity. Anakinra was effective both for flares (94%) and for long-term treatment (91%) and well tolerated. In the case of flares, 34 (6.7%) adverse effects were registered. Adverse events were more prevalent in long-term treatment.

**Conclusion:**

Anakinra was effective and safe for management of gout flares in difficult-to-treat patients. It has been used in multiple complex scenarios, such as active infections, dialysis, transplantation, chronic kidney disease, and polyarticular gout. Anakinra has also proven effective as long-term treatment, although there are more concerns about its safety.

## Highlights

- Anakinra is an effective and safe alternative in gout flares when standard therapies are contraindicated.
- Although data on long-term therapy with anakinra are scarce, the drug is effective, albeit with some safety concerns.
- Anakinra has been used in complex clinical scenarios, such as active infection, haemodialysis, and transplantation.

## Introduction

Gout is caused by the deposition of monosodium urate (MU) crystals in different tissues, leading to a chronic inflammatory response. These deposits, when intraarticular, may eventually cause acute inflammation leading to arthritis flares. Gout is the most common inflammatory arthritis in adults (1, 2), with an incidence rate ranging from 0.58 to 2.89 per 1,000 person-years, and a prevalence ranging from 1 to 6.8% in general population (3). These rates have been increasing over the last years, probably as a consequence of the aging of the population and to changes in lifestyle. Acute monoarthritis is the most characteristic clinical feature of gout and is usually the presenting symptom that rises awareness for the diagnosis of the disease, but gout is also associated with a high rate of comorbidities, especially in the elderly population. A higher prevalence of hypertension, diabetes mellitus, chronic kidney disease (CKD), and cardiovascular disease has been reported in patients with gouty arthritis compared to general population (4). This higher risk might be the consequence of the chronic inflammation and tissue damage directly related to the MU deposition.

For these reasons, the conception of the disease has evolved over the last decades: from being considered an isolated and mild arthropathy, now it is managed as a systemic inflammatory disease that associates a high disease burden with a direct impact in patients' quality of life. Nevertheless, the standard treatment for gout flares has not undergone major changes for decades. Actual treatment strategies include nonsteroidal anti-inflammatory drugs (NSAIDs), corticosteroids, and colchicine. Remarkably, these drugs are contraindicated in an increasingly number of gout patients due to the increasing comorbidities. Furthermore, among those patients in whom the standard of care is suitable, some will respond poorly and will need an alternative treatment (5). For these reasons, gout patients can constitute a challenging group when choosing a treatment strategy for the management of their arthritis flares.

It is well known that the underlying mechanism of gout clinical flares is led by the release of interleukin 1 (IL-1) by the activated NLRP3 inflammasome (6). Therefore, IL-1 has been identified as a therapeutic target for gout patients undergoing a flare. In fact, IL-1 antagonism using the IL-1β-specific antibody, canakinumab, has been approved in Europe and in the United States of America for the treatment of flares in patients in whom the standard of care is contraindicated or not well tolerated (7, 8), as well as in those who do not respond to NSAIDs and/or colchicine (9). Nevertheless, its high economic cost may limit its availability in daily clinical practice (10). As an alternative, off-label prescription of the IL-1β receptor antagonist anakinra has been reported to be effective and with an acceptable safety profile. Anakinra has been evaluated in refractory cases (9, 11–21) and in two randomized, double-blind, active-control trials (22, 23).

The main objective of the study is to perform a scoping review about characteristics, comorbidities, effectiveness and safety profile in gout patients treated with anakinra. Difficult-to-treat patients and clinical specific scenarios were described. The secondary objective is to describe the exposure and outcome of 10 cases treated with anakinra in a University Hospital.

## Materials and methods

We described the experience using anakinra for the treatment of 10 complex cases of gout patients undergoing acute arthritis flares in a teaching hospital. Then, a scoping review of the literature regarding characteristics, comorbidities, effectiveness, and safety profile of anakinra in gouty arthritis is presented.

### Case series

Patients: the catchment population comprised more than 500,000 inhabitants from an urban district in Barcelona attended in a tertiary teaching referral center (Hospital Universitari de la Santa Creu i Sant Pau, Barcelona). The study was carried out using the medical records and the electronic database from patients attended in the rheumatology outpatient clinic from January 2009 to December 2020. All patients with gouty arthritis treated with anakinra were identified and followed up for at least 1 month after their last anakinra injection to evaluate treatment response. Treatment response was defined as complete clinical resolution of the acute arthritis flare, including resolution of pain, swelling and redness, and absence of other inflammation signs. The comorbidities measured were cardiovascular disease, hypertension, dyslipidaemia, diabetes mellitus, chronic kidney disease and transplantation. The patient was considered to have the disease if it appeared in the history or anamnesis or if he/she was taking any specific treatment. All these patients were considered complex or refractory cases because, as commented before, anakinra is prescribed in our center only for acute gout flares when the standard of care failed or was contraindicated. The disease characteristics, comorbidities, response to treatment and adverse events were described.

### Scoping review

To review the characteristics, comorbidities, effectiveness, and safety profile of anakinra in gouty arthritis, a scoping review was performed following the Preferred Reporting Items for Systematic reviews and Meta-Analyses statement for Scoping Reviews (PRISMA-ScR).

#### Eligibility criteria

A search for articles in English, Spanish, and French was conducted using PubMed from January 2000 to December 2020, using MeSH terms and a free text-based search on various combinations for anakinra, IL-1 blockade, and gout (see Supplementary material). Studies that reported data regarding comorbidities, disease characteristics, effectiveness, and safety of anakinra for the treatment of gout flares were included for review. Studies carried out in animals, abstracts, conference papers, narrative reviews and editorials were excluded.

Additional references were retrieved manually by reviewing the references of the studies included. An update of the systematic research was performed before the submission of the manuscript. The search strategy is detailed in Supplementary material and the results of the update in the Supplementary Table S1.

#### Article selection and data synthesis

The citations retrieved were screened for review by C.D.T. and M.A.P. The reviewers independently ran a two-stage screening process by title/abstract and full-text reading. Data were extracted and synthesized by AG-G, SJ-N, and CD-T. Mendeley 1.19.4 software was used to manage the literature references. Articles that finally met the selection criteria were included for data extraction and synthesis.

Data was charted by SJ-N and verified by CD-T. Discrepancies in charted data were resolved by consensus discussion with the research team.

Short-term treatment (flare) was defined as injected anakinra for ≤14 days (24). Data regarding population, geographic location, outcomes, and results were recorded. A formal risk bias assessment and a qualitative synthesis were planned only if the characteristics of the studies allowed its performance.

The data were summarized according to disease characteristics, comorbidities, response to treatment, and adverse events. We also provided information about complex clinical scenarios that led to anakinra being considered the preferred treatment.

This study was approved by the ethics committee of Hospital de la Santa Creu i Sant Pau (IIBSP-ANA-2020-124) and performed in accordance with the ethical principles of the Declaration of Helsinki.

## Results

### Case series

Ten patients treated with anakinra for acute gout flare were identified in our studied population. The demographical and clinical characteristics of these patients are shown in Table 1. Nine of them were male (90%), and the age ranged from 48 to 84 years old at the moment of the prescription of anakinra, with a median age of 70.5 years. One patient presented a polyarticular disease, two of them tophaceous disease, and four of them were cataloged as a polyarticular and tophaceous disease. All of the patients had at least two comorbidities, being hypertension (all 10 patients) and CKD (seven patients) the most frequent. Among those with CKD, one was receiving hemodialysis at the time of anakinra prescription and two had previously undergone a kidney transplant. Two of the patients were hospitalized at the time of prescription, one of them undergoing an active infection (acute cholecystitis). One of the eight outpatients also presented an active urinary tract infection (UTI) at the time of anakinra prescription.

**Table 1:** Demographic and clinical characteristics of our 10 cases and anakinra treatment.

**Flare/ long- standing**	**Number of flares**	**Gender**	**Age**	**Gout characteristics**	**Comorbidities**	**Inpatient**	**Active infection**	**Previous treatments**	**Dose and length**	**Effectiveness**	**Side effects**
F	1	Male	71	Polyarticular and tophaceous	Renal transplantation, HTA, DLP, CKD	Yes	No	Corticosteroids	100 mg/24 h (3 days)	Yes	No
F	1	Male	61	Polyarticular and tophaceous	HTA, CKD, DM	No	No	Corticosteroids	100 mg/24 h (3 days)	Yes	No
F	1	Female	84	Polyarticular	HTA, DLP	Yes	Acute cholecystitis	Corticosteroids	100 mg/24 h (3 days)	Yes	No
F	2	Male	79	Monoarticular	HTA, DM, CKD, haemodialysis	No	No	Corticosteroids	100 mg/24 h (3 days)	Yes	Injection site reaction
F	1	Male	74	Polyarticular and tophaceous	HTA, CKD, DLP, Renal transplantation	No	No	Corticosteroids	100 mg/48 h (10 days)	Yes	No
C		Male	54	Tophaceous	HTA, DLP	No	No	Colchicine and corticosteroids	100 mg/24 h (3 days)	Yes	No
F	1	Male	48	Monoarticular	HTA, DLP	No	No	NSAIDs, colchicine and corticosteroids	100 mg/24 h (3 days)	No	No
F	1	Male	76	Polyarticular and tophaceous	HTA, CKD	No	No	Colchicine and corticosteroids	100 mg/24 h (3 days)	Yes	No
F	1	Male	58	Tophaceous	HTA, CKD	No	No	Colchicine and corticosteroids	100 mg/24 h (3 days)	Yes	No
F	1	Male	70	Monoarticular	HTA, CKD, DM	No	UTI	Colchicine and corticosteroids	100 mg/24 h (3 days)	Yes	No

F, flare; C, chronic; HT, hypertension; DLP, dyslipidaemia; CKD, chronic kidney disease; DM, diabetes mellitus; NSAID, nonsteroidal anti-inflammatory drug.

Treatment was clinically effective in nine of the patients. Only one mild side effect (one injection-site reaction) was recorded. No new infections were recorded after 1 month from the last anakinra injection.

### Scoping review

#### Studies' characteristics

**Figure 1:**
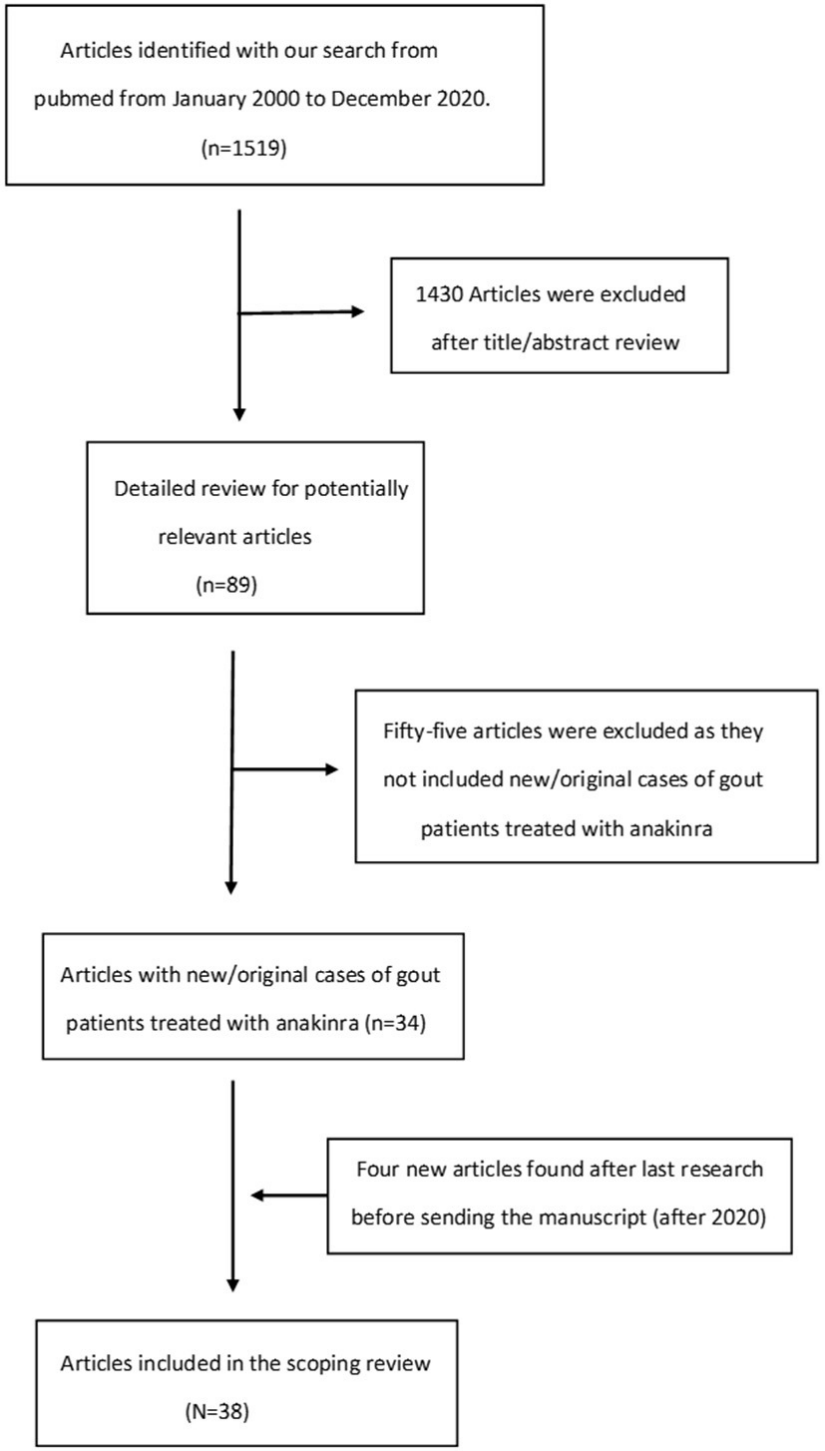
Studies selection flow chart.

The literature screening process and results are shown in Figure 1. A total of 1,519 citations were retrieved. After screening by title and abstract, 89 articles were eligible for review. After full-text reading, 34 articles including patients who fulfilled the selection criteria were included in our scoping review. From the references found in the secondary search of the bibliographies of the articles included and the update review before submission four more articles were finally included. Therefore, 38 articles were finally eligible for inclusion in our review. Two of them were RCTs (22, 23), while the other 36 studies were observational studies, case reports or case series (9, 11–21, 25–48). Due to the heterogeneity in study design and population, a scoping review rather than a systematic review was carried out to identify all type of available evidence, key concepts and knowledge gaps. Risk of bias assessment was not carried to provide an overview of the existing evidence regardless of the methodologic quality of the studies.

A total of 551 gout patients treated with anakinra were retrieved from the included studies. Anakinra was prescribed initially for the treatment of 648 gouts flares. At least 39 patients (7.1%) received anakinra for more than 7 days as long-term therapy.

#### Patients' characteristics

Four hundred and sixty-six (84%) patients were men, with a mean age of 57.9 (±10.2) years. Gout was polyarticular in 46.4% of the patients and tophaceous in 53.8%. Some of the patients presented atypical forms of the disease such as spinal gout (25, 30), autoinflammatory syndromes (27), and sternoclavicular joint arthritis (34). Most of the patients had more than one associated comorbidity among the following: hypertension (70.7%), CKD ≥3 (52.85%), DM (35.4%; Table 2).

**Table 2:** Comorbidities, demographic and clinical characteristics of gouty arthritis patients reviewed.

**Number of patients/flares; *n***	**551/648**
Male; *n* (%)	375 (83)
Age; years (± SD)	60.9 (± 10.1)
Gout treatment acute/chronic, *n* (%)	512/39 (92.9/7.1)
Polyarticular; *n* (%)	125 (47.5)
Tophaceous; *n* (%)	182 (66.9)
**Comorbidities**	
Hypertension, *n* (%)	164/232 (70.7)
CKD stage ≥ 3, *n* (%)	225/426 (52.8)
CHF, *n* (%)	150/385 (38.9)
Diabetes mellitus, *n* (%)	171/438 (35.4)
Transplant, *n* (%)	41/428 (9.7)
Dialysis, *n* (%)	14/260 (5.0)
**Previous gout flare therapy**	
Colchicine, *n* (%)	238 (43.9)
NSAIDs, *n* (%)	127 (23.4)
Corticosteroids, *n* (%)	200 (46.0)
Inpatient, *n* (%)	314 (56.9)
Flare, *n* (%)	309 (98.4)
Chronic, *n* (%)	5 (1.6)

% calculated from the total number of patients where the characteristic was reported or could be inferred. CKD, chronic kidney disease; CHF, chronic heart failure; NSAID, nonsteroidal anti-inflammatory drug.

Three hundred and fourteen patients (56.8%) were hospitalized when receiving the first dose of anakinra. The main reasons for the use of anakinra were contraindication and/or refractoriness to standard therapies. The most commonly treatments used before initiating anakinra were corticosteroids (46.0%), colchicine (43.9%), and NSAIDs (23.4%). Anakinra was the first treatment option in several patients (15, 43) (see Table 2).

#### Treatment characteristics

The administered dose of anakinra varied based on comorbidities, disease severity, and response to treatment. Daily administration of SC anakinra 100 mg was the most common pattern of prescription (81.6%) for the flares, followed by 200 mg a day (13.2%) and 100 mg every other day (4.5%). One patient received anakinra three times a week and in three cases the dose was not reported. The majority of patients received the dosage proposed by So et al. (20), i.e., subcutaneous anakinra 100 mg per day for three consecutive days. The longest treatment duration reported was 5 years (40). Patients who received anakinra every 48 or 72 h was mainly due to comorbidities or as a tapering schedule after good response to treatment, when it had to be maintained for a long time (see Supplementary Table S1).

#### Effectiveness

Although the definition of effectivity varied among the studies, anakinra was considered effective in the vast majority of the cases. Only 28 patients were reported as non-responders (5.1%), while in 25 cases efficacy data was not reported (3.9%). For the rest 598 (93.9%) of the flares, response to treatment was reported as complete or partial in a short lapse time (mainly 1–3 days). In patients with short-time relapses, retreatment with anakinra was efficacious and solved the flare. A decrease in the response to anakinra was not reported or suggested in any of the studies retrieved (see Supplementary Table S1).

### Anakinra in clinical complex scenarios

#### Active infection

Sixty-five patients were treated with anakinra for the management of a gouty flare while presenting an active infection, including critically ill patients with more than one concurrent infection at the time (15, 18, 21, 26, 36, 41) (Table 3). Most of these patients were treated with appropriate antimicrobial therapy before receiving their first anakinra dose. IL-1 blockers did not seem to affect the response to antibiotic therapy in any of the cases.

**Table 3:** Active infections at initiation of anakinra, including our case series.

**References**	**Description**
Rossi-Semerano et al. (18)	Lower airway infection
Thueringer et al. (21)	Herpes zoster *Klebsiella sp*. pneumonia, *Enterococcus sp*. bacteraemia, *Pseudomonas* UTI Candida line infection and pancreatic abscess Cellulitis Groin abscess and cellulitis Cellulitis and disseminated tuberculosis Blastomycosis pneumonia MSSA bacteraemia, septic arthritis, epidural abscess, and psoas abscess Central line infection GAS necrotizing fasciitis Disseminated multidrug-resistant tuberculosis
Liew et al. (15)	Cellulitis or abscess (*n* = 7) *Staphylococcus sp* bacteraemia (*n* = 3) *Pseudomona sp* bacteraemia *Klebsiella sp* bacteraemia (*n* = 2) Septic arthritis (*n* = 6) UTI (*n* = 5) *Clostridium difficile* colitis (*n* = 5) Pneumonia (*n* = 3) Cytomegalovirus viraemia Infectious endocarditis
Nocturne et al. (41)	H1N1 infection
Ghosh et al. (36)	Post-operative wound infection Pneumonia Sepsis
Ahmed et al. (26)	Localized infections Septic shock

UTI, urinary tract infection; MSSA, methicillin susceptible Staphylococcus aureus.

#### Organ or stem cell transplantation

Anakinra was prescribed for the treatment of 41 gout patients with a history of transplantation, as follows: stem cell (13), kidney (14, 16, 31, 40, 42), liver (15), and heart transplantation (14, 15, 17). One patient presenting chronic rejection of a renal transplantation (31) with a creatinine clearance of 14 ml/min, developed neutropenia and worsening of the renal function appeared after long-standing treatment with anakinra. One patient who underwent kidney transplantation received long-term treatment without adverse reactions (16). No other serious adverse events were reported among the rest of the patients.

#### Dialysis

Thirteen patients were on dialysis (13, 16, 21, 36). Eight patients received a daily dose, and five were treated every 48–72/h, on non-dialysis days (36). Anakinra showed efficacy and was reported to be safe in all patients.

### Safety

Overall, anakinra was well tolerated. A total of 34 (6.7%) adverse effects were reported in the flare treatment group, and most were mild or transient. As an example, seven patients (1.4%) reported injection site reactions (11, 13, 15), and five patients (0.9%) had reversible hematological disorders including three cases of leukopenia, one of neutropenia, and one with worsening of pre-existing bicytopenia (15, 21, 30). Acute infections were reported in five flares (0.9%) as follows: H1N1 virus infection (41), herpes zoster (21), severe cold (22), pulmonary abscess (17), and nosocomial pyelonephritis (16).

Among patients with a long-standing treatment with anakinra, a higher prevalence of adverse infectious was found. Seven (31.8%) infections were reported, the majority of them retrieved from the work by Ottaviani et al. (17), as follows: two *Staphylococcus aureus* tophus infections at years 1 and 4 of treatment; one *S. aureus* lung abscess after 1 month of treatment; an erysipelas infection of the leg during the second month of treatment; arthritis of the knee caused by *S. aureus* 1 year after initiation of treatment; a *Streptococcus B* urinary tract infection at the first month of treatment. Remarkably, the only case of tuberculosis reported occurred in a patient receiving long-term treatment (4 years) (42).

We specifically describe patients with active infection and patients undergoing haemodialysis or transplantation. However, some patients are treated successfully with anakinra in other refractory or difficult-to-treat conditions, such as grade >3 CKD (16, 19, 21, 29, 35, 39), severe hyperglycaemia (47), and chronic heart failure or ischaemic heart disease (9, 13, 16, 20, 21, 36, 39). In all these complex scenarios, anakinra proved to be effective and safe (see Supplementary Table S1).

## Discussion

We described our experience with refractory gout cases successfully treated with the Il-1β inhibitor anakinra in 10 patients attended in a tertiary referral hospital. We also scanned for the available literature by performing and presenting a scoping review, in which 551 gout patients treated with anti-IL1β were described. The 10 cases reported shared similar characteristics with those found in the literature, namely, patients with comorbidities for whom colchicine, NSAIDs and corticosteroids are contraindicated. Treatment with anakinra was a safe and effective option in most of the cases. The data presented provides a broad view of a real clinical practice scenario otherwise difficult to reproduce in a randomized control trial.

As the incidence and prevalence of gout are increasing, more therapeutic strategies are needed to treat recalcitrant and refractory gout flares for patients whose quality of life is otherwise severely impaired. What is more, a recent study suggests that, in gouty patients, there is an increased risk of a cardiovascular event after an attack (49). This would also argue in favors of treating attacks in a more intense way. Therefore, our study provides updated support for the use of a therapeutic alternative for a high prevalent disease with a high social and economic burden, but with a narrow therapeutic arsenal available.

As an alternative to the standard of care, canakinumab and anakinra have been reported to be effective, but several aspects limit the possibilities for the prescription of the former. First, IL-1B inhibition has been associated with a higher rate of infections. Gout patients, especially refractory ones, tend to present comorbidities and metabolic syndrome which confers a higher risk for severe infections. According to the experience summarized in our review, the treatment of an acute gout flare is usually a short-term treatment (<7 days), and therefore a medication with a shorter half-life seems more suitable for the treatment of the gout flare, avoiding the potential short-term and mid-term side effects of immunosuppression, together with other potential acute reactions. In fact, in our review, anakinra was administered for a week or less in 92.8% of the flares. Regarding this, anakinra seems more appropriate than canakinumab as their half-lives are 4–6 h and 26 days, respectively, and therefore its use seems more reasonable for frail inpatients who are prone to complications, including infections. Secondly, the high cost of canakinumab may limit its use in daily practice (10).

In terms of safety, studies including patients treated with IL-1 blockage for inflammatory arthritis have demonstrated an increased rate of infections. Our data on long-term therapy with anakinra show that 31.8% of patients developed at least one infection. This high incidence could be explained by the comorbidities of the patients studied or potential selection bias, as most of the infections are reported in the same article (17). Only one tuberculosis case was found in a patient treated with anakinra for the long term (42). Our results and those of the two clinical trials included, and according to the acute nature of the flares and the short duration of the treatment with anakinra, suggests that a possible delay on treatment initiation due to the performance of a pre-study screening and/or treatment of latent tuberculosis may not be justified. The study of Ahmed et al. (26) demonstrated that patients treated the first 48 h after the beginning of the flare had a better response.

Together with the two published trials on gout, our data suggest that anakinra is both safe and effective for the treatment of flares (22, 23), although no differences were found in terms of efficacy compared to the standard-of-care or to a single intramuscular injection of triamcinolone. Therefore, it seems reasonable that IL-1B inhibition in gout is considered when those therapeutic strategies fail or are contraindicated. Nevertheless, as anakinra has still no indication for gout, standard-of-care therapies are often used even when relative contraindications are present (10). Moreover, the number of patients with refractory gout will probably increase due to aging, and therefore complicated and refractory cases are expected to increase as well. For all these reasons, anakinra could constitute a cost-effective alternative to canakinumab.

A systematic literature review up to 2017 regarding the efficacy and safety of gout flare prophylaxis and therapy use in patients with CKD has been published by Gout, Hyperuricemia and Crystal-Associated Network (G-CAN) (50). One hundred and forty-seven patients were included from the retrieved studies. In their review they found a congress publication including complex patients (51). Eighteen gout patients received anakinra, three of them had previously undergone solid organ transplantation and seven of them had an active infection at the time of the study. All patients responded successfully to anakinra and there was only one adverse event reported, a decompensated liver failure patient presented a worsening of encephalopathy. Our results are consistent with this review, reinforcing the idea that anakinra can be a safe and efficacious option for patients with refractory gout flares.

Anakinra has also been used in other cases of crystal arthritis. It has been administered to treat calcium pyrophosphate crystal deposition (CPPD) disease, especially for the pseudogout clinical presentation (14). As CPPD crystals also activate the inflammasome, the rationale is the same as for gout. Moreover, as the prevalence of CPPD disease is also expected to increase with aging of the population, more data are needed on the use of anakinra for this condition. Perez-Ruiz et al. (52) presented a pilot study of anakinra 100 mg/week for preventing flares when urate-lowering treatment was initiated in severe tophaceous gout.

Our study is subject to a series of limitations. We have provided a cross-sectional description of our patients, and most of the data included in the scoping review come from case series (11, 14, 31, 33, 35, 37, 39, 46, 47). However, we report more than 600 flares from more than 500 patients with most of them refractory or difficult-to-treat disease. Due to the quality of the studies retrieved, which are mainly case series, a systematic review or meta-analysis could not be performed. Comparations between studies also were not possible due to the heterogeneity of the definitions including the definition of flare, refractory gout or clinical improvement.

In conclusion, the use of anakinra for the treatment of acute gout flares seems to be an effective and safe alternative to the standard of care. The dose of anakinra should be individualized depending on patient comorbidities, initial response to treatment, and experience with previous flares. High-quality control trials are needed for the standardization of the use of anakinra in gout patients, especially for refractory cases.

## Data availability statement

The raw data supporting the conclusions of this article will be made available by the authors, without undue reservation.

## Author contributions

HP, EC-A, and CD-T contributed to the conception of the work. SJ-N, AG-G, HP, EC-A, MP, and CD-T contributed in acquisition and analysis and interpretation of data. HC contributed to analysis and interpretation of data and drafting the work or revising it critically for important intellectual content. All authors revised critically the work before submission and provide approval for publication of the content.
