# Supplementary material for: Effectiveness and safety of anakinra in gouty arthritis: A case series and review of the literature
Source: Front Med (Lausanne). 2023 Jan 12;9:1089993. doi: 10.3389/fmed.2022.1089993 (PMC9877612; doi:10.3389/fmed.2022.1089993)
Supplement: Supplementary file 1 [file Table_1.DOCX]

| Supplementary table 1. Characteristics of the included studies. | | | | |
| --- | --- | --- | --- | --- |
| Study | Population | Intervention | Efficacy | Safety |
| Adler (1) | 1 patient, age 74, male. Progressive ILD with secondary PAH and CKD (unspecified autoimmune syndrome). Lumbar spine and toe gout flare. | SC Anakinra 100 mg/day for 3 days | Pain improved after the first administration, markers of clinical and serological inflammation normalized rapidly. Rare, on-demand applications of anakinra after resolution. | No safety concerns reported |
| Ahmed (2)* | 55 hospitalized patients with crystalline-induced arthritis (50 gout), mean age 60.1, 36 males. DM 21, hypertension 34, CKD 29 (52.7) AKI 14 (25.5) CAD 16 (29.1) CHF 22 (40) Obesity 21 (38.2) COPD 4 (7.3) HLD 14 (25.5) Malignancy 6 (10.9) OSA 6 (10.9) Coagulation for AF, thromboembolism 11 (20). > 2 joints affected 44,4%. | SC anakinra dosing (patients and %)   - 100 mg/d: 32 (58.2) - 100 mg twice daily: 21 (38.2) - 100 mg every other day: 2 (3.6) | - Cumulative response to anakinra (provider evaluation): 1 d 11 (20%), 2 d 26 (47.3%), 3 d 39 (70.9%), >3 d 52 (94.5%), no response 3 (5.5%).  - Cumulative response to anakinra (patient report): 1 d 17 (30.9%), 2 d 28 (50.9%), 3 d 42 (76.4%), >3 d 46 (83.6%), unknown pain score (but responded) 6 (10.9%), no response to anakinra 3 (5.5%).  No factors affecting response were found. | No safety data reported |
| Aouba 2015 (3) | 3 patients, 2 males, age 66, 71 and 72. 2 Essential arterial hypertension; 2 CKD, 1 DM, 1 neutropenia to colchicine; 1 PV, 1 obesity; 1 MGUS; 1 prolymphocytic leukemia. All polyarticular gout | SC Anakinra 100 mg/day  - for 5 days in two patients  - for 3 days in one. | Two patients (5 days) complete remission.  One patient (3 days): relapse, partial remission after anakinra 5 days, complete remission after anakinra 3 days. | One injection site reaction |
| Avram 2015 (4) | 1 patient (age 68, male), refractory acute polyarthritis, tophaceous gout. | Daily subcutaneous anakinra (100 mg per day) for 7 days, followed by one SC canakinumab 150 mg infusion | Clinical and laboratory resolution, two mild flares were successfully treated with colchicine the following months. | No safety concerns reported |
| Bacani 2009 (5) | 1 patient (age 57, male), obese, refractory acute polyarthritis tophaceous gout. | SC anakinra 100 mg daily for 3 days after the fifth and sixth rasburicase infusions | Clinical resolution of the polyarticular flare | No safety concerns reported |
| Balasubramaniam 2015 (6) | 3 patients with DM and moderate-to-severe CKD, acute gout. | SC anakinra 100 mg for 3–5 days | Immediate, transient improvement in renal function following treatment for acute gout, one of them sustained (no data about articular response) | No safety concerns reported |
| Bartov 2013 (7) | 1 patient (age 50, male), hypertension, CKD, diastolic HF, aplastic anemia, tophaceous gout, refractory acute arthritis | SC anakinra 100 mg every other day for 1 week | Complete and rapid resolution of pain and clinical evidence of diminished swelling | No safety concerns reported |
| Buenzli 2009 (8) | 1 patient, (age 73, female), acute sciatica, tophaceous gout deposits around the facet joints L3 to L5, acute phase response. | SC anakinra 100 mg daily for 3 days after local steroid infiltration of the L4–5 facet joint with partial response | Rapid clinical improvement of sciatica, normalization of the acute phase response. Subsequently maintained anakinra 100 mg three times weekly, total resolution of sciatic pain. | No safety concerns reported |
| Calvo-Aranda 2021 (9) | 1 patient, (age 56, male), smoker with a background of hypertension, hypertriglyceridaemia, hyperferritinaemia, tophi in the feet, knees, hands, elbows and shoulders. Polyarticular attacks. | SC anakinra 100 mg daily for 5 days | Attacks every 3–4 weeks during the following months despite several courses of anakinra and CSs. Weekly s.c. tocilizumab was started (162 mg), the attacks resolved after the first two doses | No safety concerns reported |
| Chen 2010 (10) | 10 patients (age 33-86, 8 males, 2 females), 2 monoarticular and 8 oligoarticular acute arthritis, 1 dyalisis. Refractory to corticosteroids | SC anakinra 100 mg per day. Mean number of anakinra injections was 3.2 for the acute flare. | 6 good responses, 3 partial responses, and 1 no response. 8 patients relapsed within 4-45 days after anakinra injection, one unknown, and one was a non-responder. | One injection site reaction |
| Desmarais 2019 (11) | 77 hospitalized patients with 98 gout flares from two hospitals OSHU: DM 48%, CKD 57%, CHF 48%, transplant 11%; 3 heart, 2 kidney.  VAPORHCS: DM 53%, CKD 62%, CHF 68%, transplant 4%; kidney. | Average number of anakinra doses (1 dose was 100 mg SC injection)   - OSSHU 4.6 (range 2–29) - VAPORHCS 3.5 (range 2–12) | OHSU: 42 flares (89%, in average 2.2 days), 4 had inadequate documentation, and 1 did not respond  VAPORHCS: 51 flares, 48 clearly responded (94%, average 1.8 days), 3 had inadequate documentation. | One patient rash on the back. Diphenhydramine prior to the third dose, tolerated a 5-day course. |
| Direz 2012 (12) | 1 patient (age 71, female). renal transplant, CTR; gouty polyarthritis affecting the knees, ankles, and elbows for 6 months | SC anakinra 100 mg for 5 days. | Decrease in pain intensity.   - Recurrence of the pain 1 week after treatment discontinuation prompted long-term daily anakinra therapy. - Anakinra stopped after 2 months due to worsening renal function requiring chronic hemodialysis. | Neutropenia after 15 days, improvement by halving the dosage (alternate days). Worsening renal function led to discontinuation. No infections. |
| Dönmez 2014 (13) | 1 patient (age 60, male), CKD, tophaceous gout. Polyarticular active synovitis; ears. Ulcerated lesions over the tophi in ankles. | SC anakinra 100 mg long term. | At the end of the first week of therapy, his synovitis regressed significantly, and he was able to walk without any support. After 4 months of therapy with anakinra and febuxostat, he had no active synovitis. | No safety concerns reported. |
| Doaré 2021 (14) | 46 patients, 31 gouty arthritis, 6 female, 21 tophaceous. Most gout patients had moderate renal failure, and frequently had cardiovascular disease, DM or arterial hypertension | SC anakinra, other biologics. | Anakinra was first biotherapy for all gout patients, and was more efficient in treating gout than CCPD: success in 90.3% vs. 35.7%, respectively (p = 0.001). Anakinra was prescribed for refractory crystal-associated arthritis or contraindications for usual treatments. | 8/30 patients had side effects, 4 discontinuations.  35 long-term-treated patients: 27 discontinuations (side effects, inefficacy, death). |
| Fedeli 2019 (15) | 1 patient (age 50, male), arthritis in left sternoclavicular joint, chronic gouty arthritis, poorly controlled type 2 diabetes, obese. | Anakinra, dosage and duration not reported. | Total remission after 6 months. | No safety concerns reported. |
| Funck-Brentano 2011 (16) | 1 patient (age 77, female), end-stage renal failure, tophaceous gout of the spine with polyarthritis, fever and biological inflammation | SC anakinra 100 mg s.c. every other day chronically. | By the 10th day of treatment, the lumbar pain, number of swollen and tender joints, and CRP improved.  Sustained improvement after 1 year of dialysis and anakinra; anakinra continued after a flare 4 days after withdrawal. | No safety concerns reported. |
| Gratton 2009 (17) | 1 patient (age 59, male), erosive tophaceous gout, type 2 DM, hypertension. Allopurinol-induced Stevens-Johnson syndrome, CKD, nephrolithiasis. Podagra. | SC anakinra 100 mg 3 times a week | Improvement, tendency to flare at the end of his dosing cycle. Anakinra dosage increased to 100 mg 4 times a week induced remission. | No safety concerns reported. |
| Ghosh (18) | 26 hospitalized patients, 22 males, 40 episodes of acute gouty arthritis. CKD 15 patients, DM 9, Congestive HF 8, Recent surgery 7, Solid organ transplantation 4, Acute infection 3, Acute leukemia 2, Steroid-associated AVN 1; ≥ 2 comorbidities 10 | SC anakinra 100 mg daily  Single dose: 4 episodes, once daily for 2 days: 4, once daily for 3 days: 17, once daily for 4 days: 1, once daily for 5 days: 3, one dose every other day for 3 days: 7, twice daily for 5: days 4. | 67% of patients responded to anakinra therapy within ≤ 1 day of treatment initiation, 18% within 48 hours, 15% 3– 6 days to achieve pain improvement. Complete resolution in 72.5% of patients within 5 days, by 10 days all but 1 patient. | 1 postoperative wound infection; possibly infected prior to anakinra. No other safety concerns reported. |
| Janssen (19) | RCT, anakinra (n = 43) or treatment as usual (free choice, n = 45). 10 hypertension, 18 cardiovascular disease, 3 DM, 4 renal disorders, 6 MSK disease (among 43 gout), 23 monoarticular, 17 oligoarticular, 3 polyarticular. | Patients with crystal-proven acute gout flare randomized to SC anakinra 100 mg 5 days + placebo (n = 43) vs. 0.5 mg up to three times daily for colchicine; 500 mg up to twice daily for naproxen or 35 mg once daily for 5 days for prednisone plus SC injection placebo once daily for 5 day (n = 45) | Improvement with no statistically significant differences between groups in mean pain, mean joint tenderness, mean treatment response scores, and mean joint swelling scores. | No serious adverse events, 15 MSK, 1 respiratory infection, 7 gastrointestinal, 2 headache, 2 fatigue, 3 pruritus, 2 paresthesia or tremor, 9 others. |
| Liew 2019 (20)* | 93 gout patients among 100 hospitalized patients with crystal arthropathies and 115 episodes of arthritis. 82% male, average age 60 years. HF 43%, CKD or ESRD 45%, DM 27%, chronic anticoagulation 22%, history of organ transplantation requiring IS 14%. 58% two or more comorbid conditions. Monoarthritis 43 episodes, oligoarthritis 56, polyarthritis 15; one systemic inflammatory response alone. Concurrent infection 34 episodes. | Indication for anakinra:   - underlying comorbidities in 84% of episodes. - failure of other therapies in 48% of episodes. - some patients both indications   Dosages:   - 100mg once 24 patients. - 100mg daily: 2 doses 13, 3 doses 52, ≥3 doses 8 - 100mg every other day: 2 doses 4, 3 doses 13, ≥3 doses 1. | 84 episodes of arthritis had partial or complete response to anakinra within four days.  66 episodes partial or complete response within one day of administration of the first dose.  Only a partial response in 7 episodes.  No response in 6.  Insufficient information in 14 episodes.  In 36 episodes, patients discharged from the hospital within four days of receiving the first dose of anakinra. | Two individuals leukopenia (one new, one with worsening of preexisting). Worsening of bicytopenia, injection site reaction, and nausea occurred in one individual each. |
| Liew 2019_2 (21) | 1 patient, age 47, male. CKD on dialysis, heart transplant, IS therapy, chronic tophaceous gout, polyarticular flare, telescoping digit. | Anakinra for the acute flare (no dose specified) | Non-reported | No safety concerns reported |
| Loustau 2017 (22) | 31 patients (24 men, mean age 72), 25 stage 4-5 CKD, 6 kidney transplantation. 60% nontransplanted and 33% transplanted had MI, stroke or peripheral artery disease. Hypertension 66% and 100% and DL 72% and 67%, respectively. | SC anakinra 100 mg daily except for 5 patients, administered every 48 or 72 hours (4 were receiving haemofiltration, administered on the days without dialysis)  Duration of AKN treatment, n (%)   - 1 to 7 days: 17 (55) - 8 to 15 days: 4 (13) - More than 15 days: 10 (32) | Anakinra was efficacious in all patients (final VAS 10 and CRP level 10 mg/L). Ten patients (32%) required prolonged treatment with anakinra. | One serious infection in one patient, 3 months after anakinra. No significant variation in renal function was observed. |
| McGonagle 2007 (23) | 1 patient (age 74, male) acute polyarthritis on chronic tophaceous gout. Membranous glomerulonephritis, hypertension, and ischaemic heart disease | SC anakinra 100 mg daily chronically | Clinical and laboratory improvement within two weeks. Residual joint tenderness, maintained the benefit from this treatment at six months | No safety concerns reported. |
| Melikoglu (24) | 1 patient (age 48, male) renal transplant due to amyloidosis caused by FMF, severe FMF attacks in spite of colchicine. Severe acute gout attacks, CKD. | Anakinra (dosage not reported) | 5-year follow-up period FMF and gout were managed successfully. | No safety concerns reported. |
| Nocturne (25) | 1 patient (age 32, male), severe juvenile-onset tophaceous gout | SC anakinra 100 mg daily for 3 days | Pain improved promptly and the polyarthritis resolved within 48 hours | Mild H1N1 influenza, onset 24h after Anakinra. |
| Ottaviani (26) | 1 patient (age 41 male), tophaceous gout, contraindication to NSAIDs and colchicine. Recurrent gouty arthritis | SC anakinra 100 mg daily, maintained for the long term (100 mg every 2 or 3 days) up to four years. | Clinical tophi decreased, complete disappearance in 2 years. | Tuberculous Cervical Lymphadenitis after 4 years of anakinra. |
| Ottaviani (27) | 40 patients (32 men; mean age 60.0) 79% clinical tophi, 92% gouty arthropathy. | SC anakinra 100 mg daily   - 3 days in 23 patients. - <15 days (6 100 mg/day, one 100 mg/2 days). - >15 days in 10 patients, followed by a spacing of the dose regimen (median duration: 5.0 months). | - Good response 36 patients (90%), - Partial response 2 patients (5%) - Non-response 2 patients (5%).   Relapse: 13 (32.5%) patients, median delay of 15 days, particularly in patients not receiving therapy to prevent acute flare (7/10 vs. 6/30, P = 0.006). No relapse with anakinra >15 days. | Seven infectious complications, mainly staphylococcal infections in six patients. One H1N1 viral infection (Reported in 18) |
| Petite 2016 (28) | 10 patients (mean age 63, 6 male), admission diagnosis of acute gout. No comorbidities reported. | SC anakinra 100 mg daily for 4 doses (3–5) during a median hospital length of stay of 7 days. | Pain scores on day 1 of anakinra were not significantly changed on discharge (6.5 vs 5.5, P = 0.9). | No safety concerns reported. |
| Rossi-Semerano 2015 (29) | 28 gout patients (mean age 57.4, 24 male, mean disease duration 1.6) among 189 patients receiving off-label anakinra for different diseases. | SC anakinra 100 mg daily, mean duration 7 days (IQR 96.5) | Partial response 6 (21.4%), total response 22 (78.6%). 18 withdrawals, 17 due to persistent remission, 1 due to on demand treatment. | 1 Pulmonary abscess in a gout patient. Overall (189), 58% patients ≥1 AE, minor injection-site reactions (39%), injection-site pain (36%), liver enzymes elevation (7%), Weight increase 11%. |
| Saad Shaukat 2020 (30) | 13 patients, hospital admission for acute gout flare colchicine-intolerant/colchicine-resistant precipitated by decompensated heart failure. Mean age 73, 5 female, Hypertension 11 (84.6%) DM 8 (61.5%) CKD 11 (84.6% ) AF 9 (69.2%) | SC anakinra 100 mg daily. Mean treatment duration was 6.54 ± 5.53 days. | All patients reported symptom improvement after 5.25 ± 3.02 days; at the time of discharge, 12 out of 13 patients (92.3%) reported symptom resolution. Anakinra was discontinued prior to discharge in all patients | No safety concerns reported. |
| Saag 2021 (31) | RCT, to assess superiority of anakinra (n=110) over triamcinolone injection (n=50). Anakinra patients: Mean age 54, 86,4%, 74.5% monoarticular, 21.8% oligo-polyarticular, 34,5% tophaceous. 50% >3 comorbidities, 42,8% 1-3, 1,8% no comorbidities. | SC anakinra 100 (56 patients) or 200 (54 patients) mg/day for 5 days, triamcinolone (40 mg in a single injection, n=55) | 301 flares were treated (214 anakinra, 87 triamcinolone). Anakinra in both doses and triamcinolone provided reduction in patient assessed pain intensity in first and subsequent flares. Anakinra performed better for physician assessment of global response to treatment, tenderness, swelling, erythema, and patient assessment of global response to treatment. | No differences between groups.  Anakinra group: majority of AEs mild, severe AEs 8 patients, serious AEs 5 patients, judged not causally related to anakinra. |
| Sharma 2019 (32) | 13 patients received anakinra for gout flare, 10 tophaceous, mean number of comorbidities 3.92, 7 DM, 12 hypertension, 8 CKD; 4 congestive HF, 8 obesity. | SC anakinra 100 mg daily:   - 1 course: 5 patients for 3 days, 3 for 5 days, 1 for 14 days, 1 for 21 days. - 2 courses: 1, 3 days courses. - 3 courses: 1, 3 days courses. - 8 courses, 1, 3-5 days courses. | Response was classified as excellent in 6 patients, substantial in 5, poor in 1, 1 lost of follow-up. 7 flares within 21 days. | Ultimate all-cause mortality 6/13 in anakinra (one sepsis, 2 CHF decompensation, 3 unknown) vs 7/52 controls. |
| Singh 2009 (33) | 1 patient (age 58, male). Poorly controlled DM, hypertension, CKD stage III, COPD, coronary artery bypass, aortic valve replacement, ICM. Gouty arthritis. | SC anakinra 100 mg daily chronically. | Clinical improvement, prednisone discontinuation within 2 months, leading to better control of DM. One gout attack since starting anakinra during a hospitalization for frequent firing of his implantable defibrillator. | No safety concerns reported. |
| So 2007 (34) | 10 patients (age 38-76), 7 acute gout, 3 chronic tophaceous gout, one with renal stones. 9 oligoarticular, 1 polyarticular. Frequent comorbidities associated. | SC anakinra 100 mg daily for 3 days. | Patient assessment of improvement in pain was 50-100% (5 patients 90-100%) within 24-48 hours. | No safety concerns reported. |
| Thueringer 2015 (35) | 13 critically ill patients with multiple comorbidities (10 ICU, 1 Burn Unit, 1 hemophagocytic lymphohistiocytosis, 1 disseminated tuberculosis), 12 males, mean age 58 years, 20 episodes of acute arthritis. 11 patients active infections | SC anakinra 100 mg daily: 5 episodes for 3 days, 2 episodes for 4 days, 7 episodes for 5 days. One flare one day, 2 flares for 2 days and 3 flares for 6 days | All patients had a significant response   - total resolution or marked improvement 50% within 24 hours,   40% by 48 hours 10% by 72 hours.  Seven patients developed recurrent flares during their hospitalization, Six received additional treatment with anakinra | 2 leukopenia, 1 previous, 1 resolution after anakinra discontinuation. 1 HZ 1 day after 6 day of anakinra. No effect on AB efficacy |
| Tran 2011 (36) | 3 patients, males aged 46, 48 and 65. All chronic tophaceous gout, one type 2 DM, hypercholesterolaemia, hypertension and obesity. | SC anakinra 100 mg daily | 3 clinical resolution, 2 polyarticular flares in 2 different patients after 4 and 5 weeks, treated with chronic anakinra (up to every 3^rd^ day) and single 6 day anakinra course respectively, with complete remission. | No safety concerns reported. |
| Van Wabeke (37) | 1 patient, 69 year, female. Tophaceoys gout, polyarticular flare. Bleeding peptic ulcer. | SC anakinra 100 mg daily 4 days | Resolution of the acute attack, free of inflammatory episodes up to one year with progressive resolution of all tophi. | No safety concerns reported. |
| Vitale (38) | 3 patients, age 50, 76 and 85, sex not reported, chronic tophaceous gout, T2D treated with oral hypoglycemic drugs, polyarticular flare. | SC anakinra 100 mg daily | Prompt and complete resolution of gouty arthritis, flare after discontinuation with good response with retreatment, better glycemic control at the 6-month follow-up visit, with no reductions in tophus volume. | No safety concerns reported. |

* data shown includes patients diagnosed with gout and other crystal arthropaties (50 gout and 5 pseudogout patients in ref.2; 93 gout and 7 CPP crystal arthritis for ref.19).

Abbreviations: AF: atrial fibrillation; AVN: avascular necrosis of bone; CKD: chronic kidney disease, COPD: chronic obstructive pulmonary disease; CTR: chronic transplant rejection, DL: dyslipidaemia, DM: diabetes mellitus; HF: heart failure; HZ: herpes zoster; ICM: ischemic cardiomyopathy; ICU: intensive care unit; ILD: interstitial lung disease; IQR: interquartilic range; IS: immunosuppression; PAH: pulmonary arterial hypertension; PV: polycitemia vera; SC: subcutaneous; MGUS: monoclonal gammopathy of undetermined significance; MI: myocardial infarction; MSK: musculoskeletal; NSAIDS (nonsteroidal anti-inflammatory drugs). Ages are expressed in years.

REFERENCES

1. Adler S, Seitz M. The gouty spine: old guy—new tricks. Rheumatology. 2017 Dec 1;56(12):2243–5.

2. Ahmed HMA, Sun D, Gaffo A. Factors Affecting Response to Anakinra in Crystalline Arthritis Flares. J Clin Rheumatol. 2022 Jun;28(4):196–200.

3. Aouba A, Deshayes S, Frenzel L, Decottignies A, Pressiat C, Bienvenu B, et al. Efficacy of Anakinra for Various Types of Crystal-Induced Arthritis in Complex Hospitalized Patients: A Case Series and Review of the Literature. Mediators of Inflammation. 2015;2015:1–7.

4. Avram A, Duarte C, Santos MJ, Papagoras C, Ritis K, Scarpioni R, et al. Identifying Patient Candidates for IL-1 Inhibition: Lessons From Real-World Cases. Joint Bone Spine. 2015 Oct;82:eS17–29.

5. Bacani AK, McCollough CH, Glazebrook KN, Bond JR, Michet CJ, Milks J, et al. Dual energy computed tomography for quantification of tissue urate deposits in tophaceous gout: help from modern physics in the management of an ancient disease. Rheumatol Int. 2012 Jan;32(1):235–9.

6. Balasubramaniam G, Almond M, Dasgupta B. Improved renal function in diabetic patients with acute gout treated with anakinra. Kidney International. 2015 Jul;88(1):195–6.

7. Bartov JB, Ali Y. Successful Use of the Interleukin 1 Antagonist, Anakinra, in a Patient With Gout, Chronic Kidney Disease, and Aplastic Anemia: Journal of Clinical Rheumatology. 2013 Dec;19(8):454–6.

8. Buenzli D, So A. Inflammatory sciatica due to spinal tophaceous gout. Case Reports. 2009 Mar 5;2009(mar02 1):bcr0720080492–bcr0720080492.

9. Calvo-Aranda E, Sanchez-Aranda FM. Efficacy of subcutaneous tocilizumab in a patient with severe gout refractory to anakinra. Rheumatology. 2021 Nov 3;60(11):e375–7.

10. Chen K, Fields T, Mancuso CA, Bass AR, Vasanth L. Anakinra’s Efficacy is Variable in Refractory Gout: Report of Ten Cases. Seminars in Arthritis and Rheumatism. 2010 Dec;40(3):210–4.

11. Desmarais J, Chu CQ. Utility of Anakinra in Acute Crystalline Diseases: A Retrospective Study Comparing a University Hospital with a Veterans Affairs Medical Center. J Rheumatol. 2019 Jul;46(7):748–50.

12. Direz G, Noël N, Guyot C, Toupance O, Salmon JH, Eschard JP. Efficacy but side effects of anakinra therapy for chronic refractory gout in a renal transplant recipient with preterminal chronic renal failure. Joint Bone Spine. 2012 Dec;79(6):631.

13. Dönmez S, Pamuk ÖN. Chronic Tophaceous Gout. J Rheumatol. 2014 Mar;41(3):554–5.

14. Doaré E, Robin F, Racapé H, Le Mélédo G, Orione C, Guggenbuhl P, et al. Features and Outcomes of Microcrystalline Arthritis Treated by Biologics: A Retrospective Study. Rheumatol Ther. 2021 Sep;8(3):1241–53.

15. Fedeli MM, Vecchi M, Rodoni Cassis P. A Patient with Complex Gout with an Autoinflammatory Syndrome and a Sternoclavicular Joint Arthritis as Presenting Symptoms. Case Reports in Rheumatology. 2020 Jan 31;2020:1–4.

16. Funck-Brentano T, Salliot C, Leboime A, Zafrani L, Servais A, Larousserie F, et al. First observation of the efficacy of IL-1ra to treat tophaceous gout of the lumbar spine. Rheumatology. 2011 Mar 1;50(3):622–4.

17. Gratton SB, Scalapino KJ, Fye KH. Case of anakinra as a steroid-sparing agent for gout inflammation. Arthritis Rheum. 2009 Sep 15;61(9):1268–70.

18. Ghosh P, Cho M, Rawat G, Simkin PA, Gardner GC. Treatment of Acute Gouty Arthritis in Complex Hospitalized Patients With Anakinra: Use of Anakinra for Acute Gouty Arthritis Patients. Arthritis Care & Research. 2013 Aug;65(8):1381–4.

19. Janssen CA, Oude Voshaar MAH, Vonkeman HE, Jansen TLThA, Janssen M, Kok MR, et al. Anakinra for the treatment of acute gout flares: a randomized, double-blind, placebo-controlled, active-comparator, non-inferiority trial. Rheumatology. 2019 Aug 1;58(8):1344–52.

20. Liew JW, Gardner GC. Use of Anakinra in Hospitalized Patients with Crystal-associated Arthritis. J Rheumatol. 2019 Oct;46(10):1345–9.

21. Liew JW, Thomason JL. Erosive Gouty Arthropathy Resulting in a Telescoping Digit: JCR: Journal of Clinical Rheumatology. 2018 May;1.

22. Loustau C, Rosine N, Forien M, Ottaviani S, Juge PA, Lioté F, et al. Effectiveness and safety of anakinra in gout patients with stage 4–5 chronic kidney disease or kidney transplantation: A multicentre, retrospective study. Joint Bone Spine. 2018 Dec;85(6):755–60.

23. McGonagle D, Tan AL, Shankaranarayana S, Madden J, Emery P, McDermott MF. Management of treatment resistant inflammation of acute on chronic tophaceous gout with anakinra. Annals of the Rheumatic Diseases. 2007 Dec 1;66(12):1683–4.

24. Melikoglu MA. Two birds with one stone: Anakinra for both gout and Familial Mediterranean fever attacks in a patient with renal transplant. Nefrología. 2020 Nov;40(6):680.

25. Nocturne G, Ora J, Ea HK, Lioté F. Influenza A H1N1 and anakinra exposure in a patient with gout. Joint Bone Spine. 2010 Jul;77(4):369–70.

26. Ottaviani S, Dieudé P. Tuberculous Cervical Lymphadenitis in a Gouty Patient Treated With Anakinra: JCR: Journal of Clinical Rheumatology. 2015 Jun;21(4):230.

27. Ottaviani S, Moltó A, Ea HK, Neveu S, Gill G, Brunier L, et al. Efficacy of anakinra in gouty arthritis: a retrospective study of 40 cases. Arthritis Res Ther. 2013;15(5):R123.

28. Petite SE. Effectiveness of Anakinra in Acute Gout: A Retrospective Review of Initial and Refractory Therapy. American Journal of Therapeutics. 2017 Sep;24(5):e633–4.

29. Rossi-Semerano L, Fautrel B, Wendling D, Hachulla E, Galeotti C, Semerano L, et al. Tolerance and efficacy of off-label anti-interleukin-1 treatments in France: a nationwide survey. Orphanet J Rare Dis. 2015;10(1):19.

30. Saad Shaukat MH, Shabbir MA, Singh S, Torosoff M, Peredo-Wende R. Anakinra for colchicine-intolerant/colchicine-resistant acute gout flare precipitated by decompensated heart failure. Ir J Med Sci. 2021 Feb;190(1):129–31.

31. Saag KG, Khanna PP, Keenan RT, Ohlman S, Osterling Koskinen L, Sparve E, et al. A Randomized, Phase II Study Evaluating the Efficacy and Safety of Anakinra in the Treatment of Gout Flares. Arthritis Rheumatol. 2021 Aug;73(8):1533–42.

32. Sharma E, Pedersen B, Terkeltaub R. Patients Prescribed Anakinra for Acute Gout Have Baseline Increased Burden of Hyperuricemia, Tophi, and Comorbidities, and Ultimate All-Cause Mortality. Clin Med�Insights�Arthritis�Musculoskelet Disord. 2019 Jan;12:117954411989085.

33. Singh D, Huston KK. IL-1 Inhibition With Anakinra in a Patient With Refractory Gout. JCR: Journal of Clinical Rheumatology. 2009 Oct;15(7):366.

34. So A, De Smedt T, Revaz S, Tschopp J. A pilot study of IL-1 inhibition by anakinra in acute gout. Arthritis Res Ther. 2007 Apr;9(2):R28.

35. Thueringer JT, Doll NK, Gertner E. Anakinra for the treatment of acute severe gout in critically ill patients. Seminars in Arthritis and Rheumatism. 2015 Aug;45(1):81–5.

36. Tran AP, Edelman J. Interleukin-1 inhibition by anakinra in refractory chronic tophaceous gout: Anakinra in refractory chronic tophaceous gout. International Journal of Rheumatic Diseases. 2011 Aug;14(3):e33–7.

37. Van Wabeke J, Dhondt E, Peene I, Piette Y. Anakinra in resistant gout: a case report. Acta Clinica Belgica. 2017 Jul 4;72(4):293–5.

38. Vitale A, Cantarini L, Rigante D, Bardelli M, Galeazzi M. Anakinra treatment in patients with gout and type 2 diabetes. Clin Rheumatol. 2015 May;34(5):981–4.
